# PLASMOpred: A Machine Learning-Based Web Application for Predicting Antimalarial Small Molecules Targeting the Apical Membrane Antigen 1–Rhoptry Neck Protein 2 Invasion Complex

**DOI:** 10.3390/ph18060776

**Published:** 2025-05-23

**Authors:** Eugene Lamptey, Jessica Oparebea, Gabriel Anyaele, Belinda Ofosu, George Hanson, Patrick O. Sakyi, Odame Agyapong, Dominic S. Y. Amuzu, Whelton A. Miller, Samuel K. Kwofie, Henrietta Esi Mensah-Brown

**Affiliations:** 1 West African Center for Cell Biology of Infectious Pathogens, College of Basic and Applied Sciences, University of Ghana, Legon, Accra P.O. Box LG 54, Ghana; lampteyeugene8@gmail.com (E.L.); jessica17appiah@gmail.com (J.O.); baofosu002@st.ug.edu.gh (B.O.); dsyamuzu.edu@gmail.com (D.S.Y.A.); skkwofie@ug.edu.gh (S.K.K.); 2 Department of Biomedical Engineering, School of Engineering Sciences, College of Basic & Applied Sciences, University of Ghana, Legon, Accra P.O. Box LG 77, Ghana; gabrielanyaele21@gmail.com (G.A.); oagyapomg@gmail.com (O.A.); 3 Department of Parasitology, Noguchi Memorial Institute for Medical Research (NMIMR), College of Health Sciences (CHS), University of Ghana, Legon, Accra P.O. Box LG 581, Ghana; gehanson@noguchi.ug.edu.gh; 4 Department of Chemistry, School of Physical and Mathematical Sciences, College of Basic and Applied Sciences, University of Ghana, Legon, Accra P.O. Box LG 56, Ghana; opsakyi@st.ug.edu.gh; 5 Department of Chemical Sciences, School of Sciences, University of Energy and Natural Resources, Sunyani P.O. Box 214, Ghana; 6 Department of Medicine, Loyola University Medical Center, Loyola University Chicago, Maywood, IL 60153, USA; wmiller6@luc.edu; 7 Department of Molecular Pharmacology & Neuroscience, Loyola University Medical Center, Loyola University Chicago, Maywood, IL 60153, USA

**Keywords:** apical membrane antigen 1 (AMA-1), rhoptry neck protein 2 (RON2), machine learning, drug discovery, malaria

## Abstract

**Objective:** Falciparum malaria is a major global health concern, affecting more than half of the world’s population and causing over half a million deaths annually. Red cell invasion is a crucial step in the parasite’s life cycle, where the parasite invade human erythrocytes to sustain infection and ensure survival. Two parasite proteins, Apical Membrane Antigen 1 (AMA-1) and Rhoptry Neck Protein 2 (RON2), are involved in tight junction formation, which is an essential step in parasite invasion of the red blood cell. Targeting the AMA-1 and RON2 interaction with inhibitors halts the formation of the tight junction, thereby preventing parasite invasion, which is detrimental to parasite survival. This study leverages machine learning (ML) to predict potential small molecule inhibitors of the AMA-1–RON2 interaction, providing putative antimalaria compounds for further chemotherapeutic exploration. **Method:** Data was retrieved from the PubChem database (AID 720542), comprising 364,447 inhibitors and non-inhibitors of the AMA-1–RON2 interaction. The data was processed by computing Morgan fingerprints and divided into training and testing with an 80:20 ratio, and the classes in the training data were balanced using the Synthetic Minority Oversampling Technique. Five ML models developed comprised Random Forest (RF), Gradient Boost Machines (GBMs), CatBoost (CB), AdaBoost (AB) and Support Vector Machine (SVM). The performances of the models were evaluated using accuracy, F1 score, and receiver operating characteristic—area under the curve (ROC-AUC) and validated using held-out data and a y-randomization test. An applicability domain analysis was carried out using the Tanimoto distance with a threshold set at 0.04 to ascertain the sample space where the models predict with confidence. **Results:** The GBMs model emerged as the best, achieving 89% accuracy and a ROC-AUC of 92%. CB and RF had accuracies of 88% and 87%, and ROC-AUC scores of 93% and 91%, respectively. **Conclusions:** Experimentally validated inhibitors of the AMA-1–RON2 interaction could serve as starting blocks for the next-generation antimalarial drugs. The models were deployed as a web-based application, known as **PLASMOpred**.

## 1. Introduction

Malaria is a life-threatening disease caused by *Plasmodium* parasites, primarily transmitted through the bites of infected female Anopheles mosquitoes [1,2,3]. Among the five *Plasmodium* species responsible for malaria in humans, *Plasmodium falciparum* is the most fatal [4,5,6,7], accounting for the majority of severe malaria cases, mostly in pregnant women and children [8,9,10]. According to the World Health Organization (WHO), malaria caused an estimated 627,000 deaths in 2022, with the vast majority of malaria-associated mortality occurring in sub-Saharan Africa and disproportionately affecting children below the age of five, who comprise 67% of malaria-related deaths [11].

The clinical manifestations of malaria occur during the blood stages of the parasite’s life cycle, where *Plasmodium* merozoites invade and exit red blood cells (RBCs) [12,13,14,15]. The process of red cell invasion is essential for the parasite’s survival, the sustenance of infection, and its transmission to mosquito vectors [16,17,18,19]. Erythrocyte invasion involves multiple highly coordinated steps, culminating in the formation of a tight junction, a transient but essential structure that acts as a molecular anchor between the merozoite and the host erythrocyte [20]. The tight junction formation begins with the interaction between Apical Membrane Antigen 1 (AMA-1), located on the merozoite surface, and Rhoptry Neck Protein 2 (RON2), which is secreted by the parasite and embedded into the RBC membrane [21,22]. Following egress from liver cells and entry into the bloodstream, the merozoite attaches to RBC receptors and quickly reorients itself to position its apical end containing the rhoptry organelles necessary for invasion toward the RBC membrane [23,24,25]. This is followed by an interaction between the Reticulocyte Binding Protein Homologue 5 (PfRH5) and host cell surface protein Basigin (CD147) [26,27,28], which causes the release of rhoptry proteins into the RBC cytoplasm [29]. Among these rhoptry proteins, RON2 integrates into the RBC membrane, creating an extracellular loop that binds specifically to a hydrophobic cleft on AMA-1 [30].

The interaction between AMA-1 and RON2 is pivotal in stabilizing the initial contact between the parasite and the host cell. It provides the structural framework for the tight junction, a junctional complex that migrates along the surface of the merozoite as the parasite invades the RBC [31,32]. This movement is powered by the parasite’s actomyosin motor, which generates the mechanical force needed to penetrate the RBC membrane [20,33]. Other rhoptry proteins, such as RON4 and RON5, contribute to stabilizing the tight junction by interacting with the host cell’s cytoskeleton, ensuring the parasite’s successful invasion [29,30].

Although artemisinin-based combination therapies (ACTs) have significantly reduced malaria morbidity and mortality, the emergence of artemisinin resistance underscores the urgent need for new therapeutic strategies [34,35]. Additionally, various malaria vaccines have been developed to combat the spread of malaria; however, these vaccines tend to show very low efficacy against the parasite, particularly *Plasmodium falciparum* [36,37]. This highlights the need for innovative drug discovery approaches. Notably, the AMA-1–RON2 interaction is indispensable for the invasion process, as it lacks alternative pathways, making it an ideal therapeutic target [21,22]. Disrupting this interaction would prevent tight junction formation, effectively halting the merozoite’s entry into RBCs and breaking the parasite’s life cycle, offering a promising approach for malaria drug discovery [22,29].

Experimental techniques such as quantitative high-throughput screening (qHTS) and structure–activity studies have been used to identify small molecule inhibitors of the AMA-1–RON2 interaction [21,22]. However, these methods are time-consuming, costly, and require significant expertise [38]. Computational approaches, particularly machine learning (ML), provide a faster, cost-effective alternative for identifying promising drug candidates [39,40]. Several studies have been conducted using ML in the drug discovery process of various diseases [41,42,43,44,45]. In the field of malaria, ML has been applied in the early diagnosis of malaria based on clinical information [46]. Considering the AMA-1–RON2 complex, ML has been applied to develop models to predict small molecule inhibitors of the AMA-1–RON2 invasion complex [47]. Also, ML has been applied in a study that designed chemical inhibitors of the hydrophobic cleft of the AMA-1 protein in *Toxoplasma Gondi* [48]. Another study developed QSAR (Quantitative Structure–Activity Relationship) models for predicting antimalarial molecules targeting the *Plasmodium falciparum* apicolast organelle [49]. Although. there have been major strides in the application of ML in the prediction of antimalarial compounds, there is still the need to explore other models to consolidate the large endeavor towards the early prediction of potential small molecule inhibitors of the AMA-1–RON2 invasion complex.

This study applied supervised ML techniques to predict small molecule inhibitors of the AMA-1–RON2 interactions. By leveraging data from qHTS, various ML models such as Random Forest (RF), Gradient Boost Machines (GBMs), CatBoost (CB), and AdaBoost (AB) were trained and evaluated. A Support Vector Machine (SVM) was also developed to assess how other algorithms perform in relation to the tree-based models. The models were validated to assess their efficiency to ensure their robustness. The top-performing models were deployed as a web-based application called PLASMOpred to aid in predicting antimalarial compounds to support the identification of novel therapeutic compounds.

## 2. Results and Discussion

### 2.1. Data Collection and Pre-Processing

The dataset obtained via PubChem (AID 720542) was screened using qHTS to evaluate compound activity [50,51,52]. Compounds were categorized as active (scores 40–100), inconclusive (scores 1–39), or inactive (score 0) [53]. The final working dataset included 738 active compounds and 356,551 compounds labeled as inactive, with the compounds annotated as inconclusive removed from the dataset. The input and output data were in the canonical SMILES format, and contained no duplicates or null values. The activity outcomes were converted into binary labels, with active represented as 1 and inactive as 0 [51,54,55]. To evaluate the models on new data, a validation dataset comprising 20 active and 20 inactive compounds was created by randomly selecting these compounds from the processed dataset. This validation dataset was used for the final evaluation of the models; hence, they were not included in the training or testing phases. To manage computational complexity and address the extreme imbalance between active and inactive classes, the dataset size was reduced to 2000 compounds by randomly reducing the inactive class using a random undersampling technique [49]. This final dataset comprised 718 active compounds and 1282 inactive compounds, maintaining the integrity of the classification task, preventing models from being biased to the majority class.

### 2.2. Descriptor Generation and Feature Engineering

Morgan fingerprints were generated using RDKit 2024.9.4 to encode molecular structures into binary vectors, capturing chemical features within a defined radius [56,57]. The Morgan fingerprint generated 2048 distinct features for each compound, enabling ML models to effectively analyze the molecular structures and identify key patterns associated with the activity [56,57,58,59]. To further enhance model performance, a variance threshold with a threshold of 0.1 was applied to remove features with low variance, such as columns containing all zeros or all ones. These features may contribute little to model learning and prediction due to their lack of variability [60]. For this study, only one feature was filtered out based on low variance, reducing the dimensionality of the dataset to 2047 features.

### 2.3. Data Splitting and Data Balancing

The processed dataset was split into training and test sets in the ratio 80:20, with a random state parameter of 18 to ensure reproducibility. The split resulted in a training dataset of 1568 compounds and a testing dataset of 392 compounds, each with 2047 features. Using the same training dataset at a random state set at 18 across ML models allowed for comparisons of performance metrics and evaluation. To address the class imbalance between active and inactive compounds, the Synthetic Minority Oversampling Technique (SMOTE) was applied to the training dataset. SMOTE generates synthetic samples of the minority class (active compounds) by interpolating between existing active compounds, thereby balancing the class distribution [61,62,63]. This approach enhances the model’s ability to accurately predict active and inactive compounds without bias toward the majority class. By applying SMOTE, the size of the training dataset increased to 1998 compounds, with 430 synthetic active compounds added to the dataset.

### 2.4. Development and Evaluation of Machine Learning Models

This study utilized five ML models comprising RF, GBMs, CB, AB, SVM, with an emphasis on tree-based models for the effective handling of class imbalances [64,65,66]. Hyperparameter tuning with grid search optimized each model by identifying the best hyperparameter combinations [67,68,69]. For the RF model, the optimal parameters included a maximum number of features set to 5, the number of estimators set to 185, and a random state value of 2. The GBMs model achieved its best performance with 150 estimators, a learning rate of 0.2, a maximum depth of 9, and a random state value of 5. For the CB model, the best hyperparameter combination consisted of 185 iterations, a depth of 5, a learning rate of 0.1, and a random seed value of 2. The AB model performed optimally with a base estimator configured with 185 estimators, a learning rate of 1.0, and a random state value of 2. The SVM model achieved its best results using a polynomial kernel with a degree of 3, a coefficient value of 1.0, and probability set as True.

**Figure 1:**
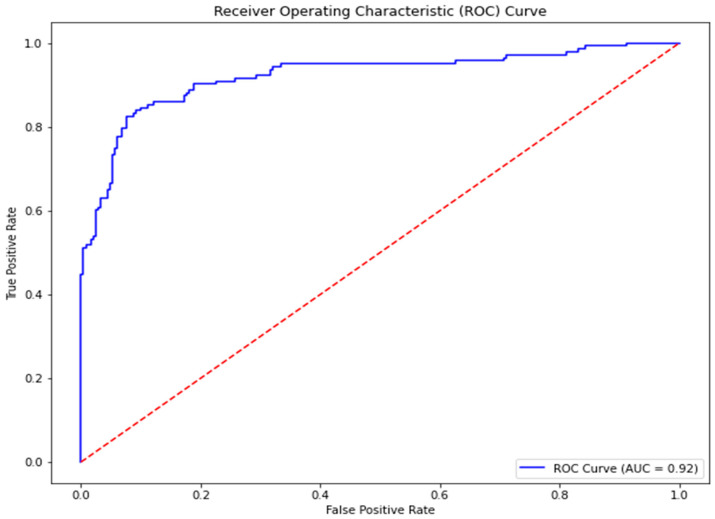
Receiver Operating Curve for the best-performing model, GBMs. The ROC-AUC obtained was 0.92.

The models were evaluated using the following metrics: specificity, sensitivity, F1-score, precision, accuracy, and the receiver operating characteristic—area under the curve (ROC-AUC) (Table 1). Sensitivity generally measures the model’s ability to correctly identify active compounds (true positives) among all actual active compounds [70,71,72]. Among the models, CB achieved the highest sensitivity with a score of 0.87, indicating its strong performance in detecting active compounds. This was followed by GBMs and RF with scores of 0.85 each. The SVM model performed better than AB, with a score of 0.86 compared to 0.77. The lower sensitivity of AB suggests it may be less effective at identifying active compounds, potentially missing some true positives. Specificity measures the model’s ability to correctly identify inactive compounds (true negatives) among all actual inactive compounds [70,71,72]. AB achieved the highest specificity with a score of 0.92, demonstrating its potential effectiveness in classifying inactive compounds. GBMs, CB, and RF also performed strongly, with scores of 0.90, 0.88, and 0.88, respectively. The SVM model, however, scored the lowest with 0.80. Precision indicates the proportion of true positive predictions among all positive predictions made by the model [73]. AB achieved the highest precision with a score of 0.85, followed closely by GBMs with 0.84. RF and CB scored 0.81 and 0.80, respectively, showing comparable results. SVM had the lowest precision, scoring 0.71, which suggests a higher rate of false positives compared to the other models, in line with its low specificity. The F1-score is the harmonic mean of precision and sensitivity, balancing these two metrics to provide a single measure of the model’s performance [73,74,75]. CB and GBMs achieved an F1-score of 0.84, while RF scored 83, indicating their strong overall balance between precision and sensitivity. AB and SVM scored slightly lower, with scores of 0.81 and 0.78, respectively. Accuracy reflects the proportion of correctly predicted compounds (active and inactive) out of the total number of predictions [76,77]. GBMs achieved the highest accuracy with a score of 0.89, followed by CB with 0.88 and RF with 0.87. AB scored 0.86, while SVM had the lowest accuracy with a score of 0.82, demonstrating comparatively weaker overall performance. The ROC-AUC provides a measure of the model’s ability to distinguish between active and inactive compounds across different classification thresholds [78,79]. CB and AB achieved the highest ROC-AUC scores, both at 0.93, highlighting their superior classification capability. GBMs and RF also performed strongly with scores of 0.92 and 0.91 (Figure 1), respectively. The SVM model scored slightly lower, with 0.90. The performance of the models is comparable to that in other similar ML studies [46,47,48,49] In another study, the RF model, which had the best performance, achieved an accuracy score of 0.93 and an ROC-AUC score of 0.84 [46]. However, other metrics, such as the F1-score and precision, were low at 0.19 and 0.3, respectively, probably due to limitations data [46]. The best-performing model in the study that identified inhibitors of the AMA-1–RON2 interaction, using the PubChem AID720542, was the RF model, which achieved specificity and ROC-AUC scores of 0.8 and 0.86, respectively [47]. This study showcased the performance of the RF model with a sensitivity of 0.85 and a ROC-AUC score of 0.91. The differences in results might be attributed to the implementation of random undersampling and SMOTE to balance the active and inactive classes in this study.

In the study that developed QSAR models to predict antimalarial molecules targeting the apicoplast organelle of *Plasmodium falciparum*, the best-performing model was also the RF model, which achieved accuracy, ROC-AUC, and sensitivity scores of 0.87, 0.88, and 0.86, respectively [49]. These results are comparable to those of this study. Additionally, a SVM model achieved accuracy, AUC-ROC, and sensitivity scores of 0.83, 0.88, and 0.82, respectively [49], which are also comparable to the SVM model in this study (Table 1). Although, the study predicted potential antimalarial molecules focusing on a different target [49], the methods used, such as undersampling, to handle class imbalance were consistent with those in this study. A key difference was the use of Recursive Feature Elimination (RFE) [49], whereas this project employed a variance threshold.

GBMs outperformed other models due to its ability to capture complex feature interactions, iteratively refine weak learners, and assign higher weights to misclassified samples, improving generalization. The performance of SVM was likely due to its sensitivity to large datasets and imbalanced classes. Unlike tree-based models, which adaptively handle features, SVM relies on finding an optimal decision boundary, which can be challenging when dealing with overlapping class distributions.

### 2.5. Validation of Machine Learning Models

**Figure 2:**
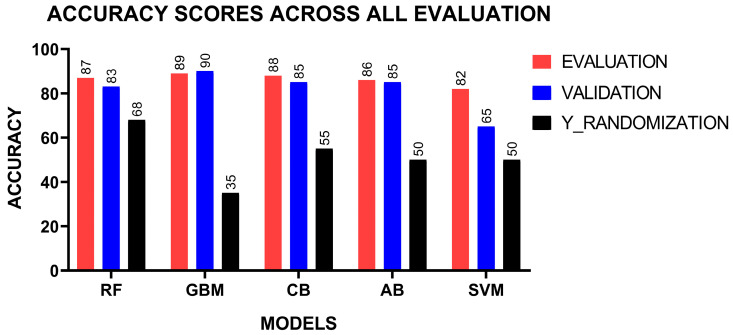
Assessment of models on the test data using validation, held-out data, and y-randomization.

The models were validated using the 40 compounds which were held back at the beginning of the study. This dataset comprised 20 active and 20 inactive compounds. Similar to the training and testing phases, Morgan fingerprints were computed from the canonical SMILES as input features, while the activity outcome served as the output. Each model was validated on this new dataset and evaluated based on accuracy scores. Additionally, the y-randomization test was performed on the validation dataset, where the activity outcome labels were shuffled. The expected outcome of this test is a reduced model performance, which validates the models’ ability to make accurate predictions while ruling out overfitting [80,81]. The degree of performance reduction depends on the extent of shuffling for each specific model. The performance of the models were comparable during validation to their initial evaluation on the test dataset (Figure 2). This perhaps highlights their capability to maintain high performance on previously unseen data. The GBMs achieved the highest accuracy score of 0.90, followed by CB and AB, which had 0.85 (Table 2). RF achieved an accuracy score of 0.83 whilst SVM, had a lower accuracy score of 0.65. Perhaps this supports the hypothesis that tree-based methods are robustly suited for this study. The accuracy scores from the y-randomization test decreased for all the models. This reduction may highlight the effectiveness of the models and decreasing overfitting.

### 2.6. Model Deployment

**Figure 3:**
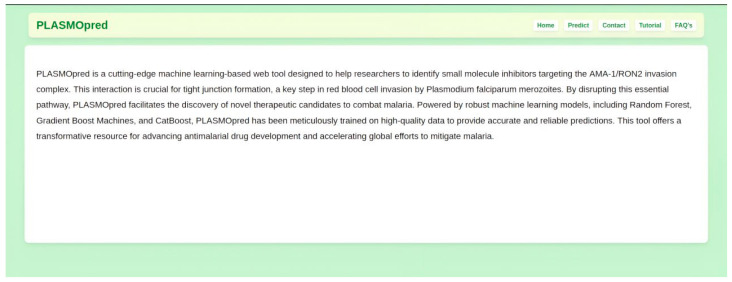
A display of the homepage of the deployed web application PLASMOpred.

**Figure 4:**
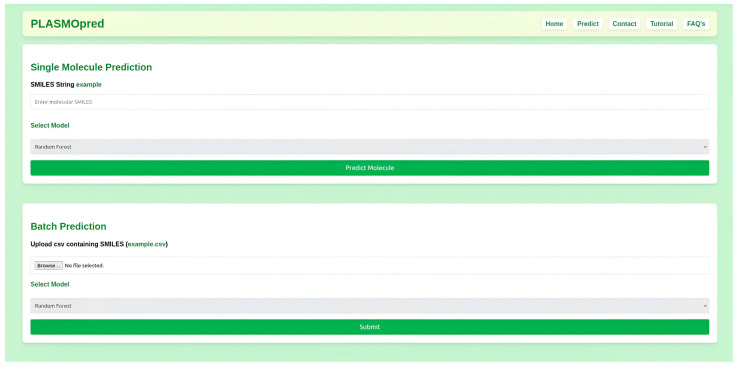
A graphical user interface of PLASMOpred for the prediction of antimalarial activity.

**Figure 5:**
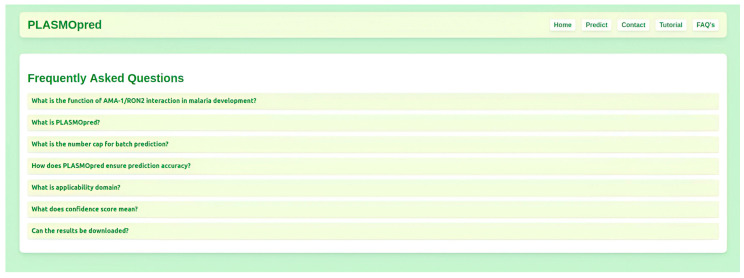
Frequently asked questions page of the deployed web application PLASMOpred.

For the top three performing predictive models, which were RF, GBMs, and CB, a user-friendly platform was provided that allows researchers to input chemical structural data, such as SMILES, and receive results instantly (Figure 3, Figure 4 and Figure 5). The web deployment was built with scalable technologies, ensuring that it can handle many simultaneous users while maintaining computational efficiency. Additionally, the application includes features like data visualization, which allows users to interpret the results of the model in a more accessible format. This deployment enhances the accessibility of the model. The web application is accessible at http://197.255.126.13:8081 (accessed on 14 April 2025).

## 3. Applicability Domain

**Figure 6:**
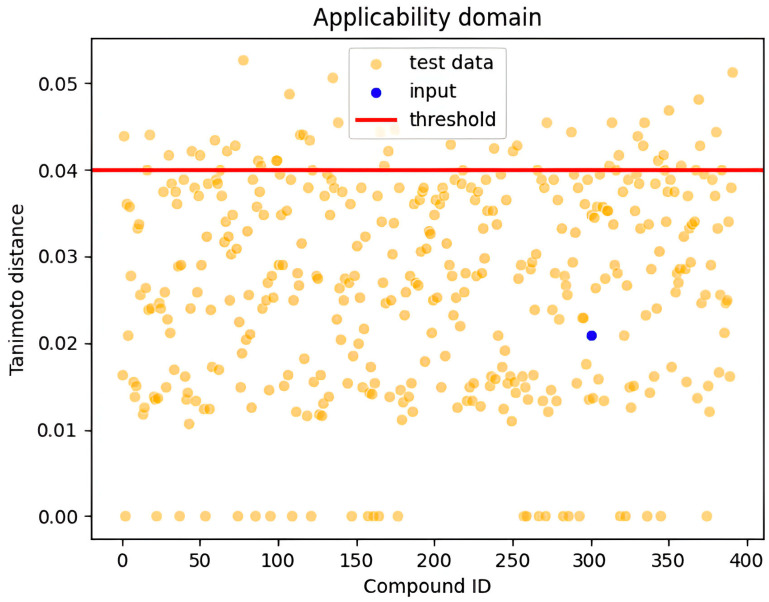
Visualization of the applicability domains showing all test data points and the input point.

In this study, the applicability domain (AD) was defined using the Tanimoto distance with a threshold value set at 0.04. The applicability domain defines the response and chemical structure spaces in which a model makes predictions with a given reliability (boundaries, likelihood, applicability, and reliability) [82,83]. Common approaches for determining the applicability domain examine molecular classes, features, and agreement in both chemical and response domains; a typical measure is based on distances within these domains [82,83]. The Tanimoto distance is a widely used metric in chemoinformatics to quantify the similarity between the feature vectors of new samples and the training data [82,83]. Samples with a Tanimoto distance below the threshold of 0.04 were considered to fall within the applicability domain, where the model showed reliable predictive performance [82]. In contrast, samples with a Tanimoto distance exceeding this threshold were deemed outside the applicability domain, and the model’s predictions for these samples were less reliable. The threshold of 0.04 was chosen based on the model’s performance on validation data, ensuring that predictions remain valid for most practical scenarios while highlighting the risk of inaccuracy for samples with significant differences from the training set. This approach allows for a controlled balance between model confidence and generalizability [82,83]. Figure 6 illustrates the applicability domain of our samples, showing the distribution of Tanimoto distances for the training set, with a vertical line at 0.04 indicating our selected threshold; the region below the threshold represents compounds with reliable predictions, while those above are compounds for which predictions should be treated with caution.

The Tanimoto distance is calculated using the following:

(1) $$\mathrm{Tan}\mathrm{imoto}\;\mathrm{Distance}=1-\frac{A\cap B\;}{A\cup B\;}=\frac{|A\cap B|\;\;\;}{|A|+|B|-|A\cap B|}$$

where *A* and *B* are two sets (or feature vectors), and ∣*A*∩*B*∣ represents the number of common elements between the sets, while ∣*A*∣ and ∣*B*∣ are the sizes of the individual sets.

## 4. Materials and Methods

### 4.1. Data Collection

**Figure 7:**
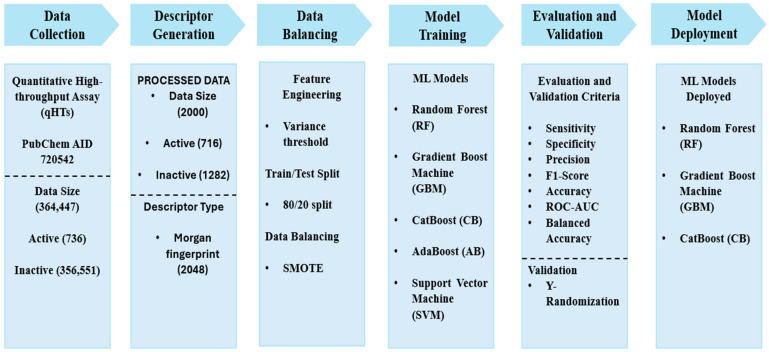
Methodology schema highlighting the project pipeline. It shows information on data collection, descriptor generation, data balance, model training, evaluation, validation and deployment.

The dataset used for the classification of inhibitors was sourced from PubChem with AID 720542. The assay was performed by the National Center for Advancing Translational Sciences (NCATS) within the National Institute of Health (NIH) in the United States and comprised 364,447 compounds generated through qHTS. The purpose of the qHTS assay was to identify inhibitors of the AMA-1–RON2 interactions towards the development of antimalarial drug leads [53]. The qHTS is an advanced screening technique that tests compounds over a range of concentrations to generate detailed dose–response curves [50,51]. Compounds in the dataset were classified based on their activity score obtained through efficacy, AC50, and curve class. Activity scores, recorded as PubChem activity scores, ranged between 40 and 100 for active compounds, 1 and 39 for inconclusive compounds, and 0 for inactive compounds. Fit_LogAC50 was employed to scale scores within each curve class, providing a relative ranking of compound activity [53]. Out of the total dataset, 738 compounds were classified as active, 7158 were inconclusive, and the remaining 356,551 compounds were inactive (Figure 7). Before generating descriptors, the dataset was pre-processed to ensure accurate and reliable models. The PubChem canonical SMILES column was used as the input, which contains the SMILES notation for the compounds, and the PubChem activity outcome column as the output, which contains the activity outcome for each compound. The activity outcome was converted from strings to integers with 1 (active) and 0 (inactive) [51], while inconclusive entries were excluded, leaving only active and inactive compounds in the dataset. To create a validation dataset, 20 active and 20 inactive compounds were randomly held out from the processed dataset. Given the large dataset size, the number of inactive compounds was randomly downsampled to create a combined dataset of 2000 compounds using the Numpy version 1.21.4 Python library, consisting of 718 active and 1282 inactive compounds, to manage the high computational demand and address the extreme class imbalance. This reduced dataset was used for training and testing the ML models.

### 4.2. Descriptor Generation and Feature Engineering

Morgan fingerprints were computed for each compound resulting in 2048 distinct features. These fingerprints, which encode molecular structures as mathematical representations, serve as input features for the models [56,57]. To improve performance and minimize redundancy, a variance threshold with a threshold of 0.1 was applied to remove features with low variance [60]. The dataset was then split into an 80:20 ratio, as this provided the optimal performance of the models, with 80% of the data allocated for training and 20% for testing [84,85]. To address the class imbalance in the training data, the SMOTE was employed [61,86]. SMOTE was applied to the active compounds, the minority class, generating 430 synthetic samples to ensure a balanced dataset for training.

### 4.3. Development and Evaluation of Machine Learning Models

Five ML models comprising RF, GBMs, CB, AB and SVM were developed as webservers for the prediction of small molecule inhibitors [87,88,89]. The tree-based methods (RF, GBMs, CB, AB) were hypothesized to better fit the dataset due to their ability to handle complex feature interactions and imbalances effectively [64]. The SVM model was included as a model with a different classification mechanism. Each model was built and optimized using unique hyperparameters tailored to its architecture. RF was constructed with parameters such as maximum number features, number of estimators, and random state [90]. GBMs were trained using number of estimators, maximum depth, learning rate, and random state [91,92,93]. For CB, the parameters included iterations, depth, learning rate, and random seed [94,95]. The AB model was trained with number of estimators, learning rate, and random state [96,97,98]. For the SVM, the training data were scaled due to the algorithm’s reliance on distance-based metrics [99,100], and the model was built using parameters including kernel, degree, probability, and coefficients [101,102,103]. Hyperparameter optimization was performed for each model using a grid search to identify the best combination of parameters to ensure optimal performance [67]. The RF, GB, and AB models had the number of estimators set within a range of 50 to 300 with an interval of 5, while the maximum depth parameters ranged from 3 to 10 for all models that utilized it. The CB, AB, and GBMs models incorporated the learning rate parameter, which was set within a range of 0.01 to 0.3. For RF, the minimum samples leaf parameter ranged from 1 to 6 with an increment of 1, while the minimum samples split parameter ranged from 2 to 11, increasing by 2 at each step. For the SVM, the degree parameter was set within a range of 2 to 5 with an increment of 1, while the coefficient parameter ranged from 0.0 to 2.0. The optimized models were evaluated using several classification metrics, including specificity, sensitivity, F1-score, precision, accuracy, and ROC-AUC [78,104,105,106]. These metrics (Equations (2)–(6)), provided a comprehensive assessment of the models’ performance and suitability for the prediction task.

(2) $$\mathrm{Accuracy}=\frac{TP+TN}{TP+TN+FP+FN}$$

(3) $$\mathrm{Precision}=\frac{TP}{TP+FP}$$

(4) $$\mathrm{Sensitivity}=\frac{TP}{TP+FN}$$

(5) $$\mathrm{Specificity}=\frac{TN}{TN+FP}$$

(6) $$F1-\mathrm{score}=\frac{2\times Precision\times Sensitivity}{Precision+Sensitivity}$$

Note: *TP*, *TN*, *FP*, and *FN* represent true positives, true negatives, false positives, and false negatives, respectively.

### 4.4. Validation of Machine Learning Models

The optimized ML models were validated using a held-out dataset of 20 active and 20 inactive compounds. This dataset was entirely excluded from the training process and used to evaluate the models’ performance on new, unseen data. The held-out validation aimed to assess the models’ generalizability and robustness in predicting the activity of novel compounds. To further validate the models and ensure there was no overfitting, a y-randomization test was conducted. In this test, the activity outcomes (outputs) were randomly shuffled while keeping the descriptors (inputs) intact [80,81]. The models were then re-evaluated on this randomized dataset, and any significant decrease in performance confirmed the models’ reliability and ruled out overfitting. An applicability domain analysis was also carried out using the Tanimoto distance applicability domain with a threshold of 0.04 to assess the chemoinformatics space where the models can make accurate predictions.

### 4.5. Model Deployment

The model was successfully deployed as a web application, making it accessible for users to perform predictions. The front end of the web application was built using Hypertext Markup Language (HTML) and Cascading Style Sheet (CSS), which together ensure an intuitive and visually appealing user interface. HTML provides the structural framework of the application, defining how content like text, forms, and buttons are organized on the web page, while CSS enhances the visual design by controlling the layout, colors, fonts, and overall styling of the interface. For the back end, Python version 3.10.0 and Flask were used to create the logic and functionality of the application. Python was used for handling the core predictive computations, including pre-processing user-inputted data and running the ML models [107]. Flask facilitated the development of the application’s back end, enabling smooth communication between the front end and the back end and managing Hypertext Transfer Protocol (HTTP) requests and responses [108]. Additionally, JavaScript was incorporated to enhance the interactivity of the application, enabling dynamic features such as updates and interactive components, thereby improving the user experience [109].

## 5. Limitations

This study employed random undersampling to address the significant class imbalance and enhance computational efficiency. Random undersampling reduces the size of the majority class (inactive compounds) by randomly removing a portion of its instances. This technique is widely used in ML to balance datasets and prevent models from being biased toward the majority class. Additionally, it helps mitigate overfitting by ensuring that the classifier does not learn a decision boundary heavily skewed by the majority class [110]. However, a key limitation of this approach is the potential loss of valuable information. Since the process randomly removes inactive compounds, it may discard instances that contain critical patterns or edge cases necessary for distinguishing between active and inactive compounds. This loss can impact the model’s ability to generalize well to unseen data. The predictions of the models need experimental validation to corroborate the computed biological activity. In future, larger datasets devoid of class imbalances maybe used to optimize and enhance the performance of the models.

## 6. Conclusions

The study developed five ML models comprising RF, GBMs, CB, AB, and SVM using bioactive datasets sourced from the PubChem database to predict potential small molecule inhibitors of the AMA-1–RON2 interaction, which is essential for erythrocyte invasion by *Plasmodium falciparum.* Morgan fingerprints of the compounds were calculated, resulting in 2047 features after variance filtering, which were used to train the predictive models. GBMs emerged as the best model, with an accuracy of 0.90 and an ROC-AUC score of 0.92. CB and AB also had ROC-AUC scores of 0.93 each. The models were validated using a held-out dataset and y-randomization tests, confirming their robustness and ruling out overfitting. The optimized models were deployed as a web application, PLASMOpred, providing a user-friendly interface to support the prediction of AMA-1–RON2 inhibitors. These robust and efficient models enhance the ability to identify putative inhibitors, offering significant contributions to computer-aided antimalarial discovery. 

## Figures and Tables

**Table 1:** Evaluation of the optimized ML models for RF, GBMs, CB, AB and SVM.

METRICS	MODELS				
	RF	GBMs	CB	AB	SVM
Sensitivity	0.85	0.85	0.87	0.77	0.86
Specificity	0.88	0.90	0.88	0.92	0.80
Precision	0.81	0.84	0.80	0.85	0.71
F1-score	0.83	0.84	0.84	0.81	0.78
Accuracy	0.87	0.89	0.88	0.86	0.82
ROC-AUC	0.91	0.92	0.93	0.93	0.90

**Table 2:** Evaluation of the optimized ML models on held-out data.

METRICS	MODELS				
	RF	GBMs	CB	AB	SVM
Sensitivity	0.80	0.85	0.85	0.80	0.50
Specificity	0.85	0.95	0.85	0.90	0.80
Precision	0.84	0.94	0.85	0.88	0.71
F1-score	0.82	0.89	0.85	0.84	0.59
Accuracy	0.83	0.90	0.85	0.85	0.65
ROC-AUC	0.80	0.85	0.85	0.80	0.50

## Data Availability

All data utilized and obtained for this project are provided within the manuscript.
